# The Effectiveness of Targeted Electrical Stimulation *via* Cochlear Implant on Tinnitus-Perceived Loudness

**DOI:** 10.3389/fnins.2022.885263

**Published:** 2022-06-24

**Authors:** Walter Di Nardo, Tiziana Di Cesare, Angelo Tizio, Gaetano Paludetti, Anna Rita Fetoni

**Affiliations:** ^1^Dipartimento di Scienze dell’Invecchiamento, Neurologiche, Ortopediche e della Testa-Collo, UOC di Otorinolaringoiatria, Fondazione Policlinico Universitario A. Gemelli IRCCS, Rome, Italy; ^2^Dipartimento di Neuroscienze, Sezione di Audiologia, Universitá Federico II, Naples, Italy

**Keywords:** cochlear implant, tinnitus, intracochlear electrical stimulation, cochlear regions, pitch match

## Abstract

**Introduction:**

The cause of tinnitus improvement in cochlear implant (CI) users is not understood. On the basis that a spatially limited dysfunction in the auditory pathway could cause tinnitus, we used single-channel stimulation to evaluate any variation of tinnitus-perceived loudness and identify the cochlear regions involved.

**Materials and Methods:**

It was an observational prospective case-crossover study. After the first mapping, 21 adults with unilateral CI and chronic tinnitus expressed their tinnitus loudness based on the Visual Analog Scale (VAS) score (0–10) at baseline (L^0^), during a 10 s single-channel stimulation with C-level of electric current (L^1^) and 30 min after CI activation (L^2^). Tinnitus reduction [R*^T^* = (L^0^ – L^1^) × 100/L^0^] > 50% was considered significant. VAS outcomes were compared between baseline (L0) and (each) single-channel stimulation (L1) to find the channel with the greatest R*^T^* (suppressive channel-SC), whose frequency range revealed the cochlear region involved. Seven patients with asymmetric hearing loss underwent the pitch-matching test to identify the actual frequency evoked by the SC. We compared selective (L^1^) and non-selective (L^2^) intracochlear stimulation using paired *t*-test. Preoperative Tinnitus Handicap Inventory (THI) score was compared with those at 1, 6, and 12 months with paired *t*-tests to evaluate long-term tinnitus perception.

**Results:**

We observed a significant reduction of tinnitus loudness during the experimental procedure [L^0^ (6.4 ± 2.4) vs. L^1^ (1.7 ± 2.7), *p* = 0.003]. A total of 15/21 patients (71.4%) had a significant (R*^T^* > 50%) and selective improvement, reporting a mean L^1^ of 0.4 ± 2.0 (*p* = 0.0001). In 10/15 (66.6%) patients, the SC was in the apical turn, within 1,000 Hz; in 5/15 patients (33.4%) within 4,000 Hz. The cochlear region 125–313 Hz was the most affected by tinnitus improvement (*p* = 0.0074). Targeted stimulation was more effective than non-selective stimulation [L^1^ vs. L^2^ (4.3 ± 2.5), *p* = 0.0022]. In 3/7 patients, the perceived pitch did not fall within the SC frequency ranges. All patients with selective attenuation described tinnitus as monotone. Patients with non-selective attenuation had polyphonic tinnitus and better THI results after 1 year.

**Conclusion:**

Targeted intracochlear electrical stimulation improved chronic tinnitus perception, especially in monotone tinnitus, and the apical region was mainly involved. Our results provide new insights into the pathophysiological mechanisms of tinnitus and targets for innovative therapeutic strategies.

## Introduction

Subjective tinnitus, consisting of the perception of sounds without a corresponding acoustic stimulus, is a very common and disabling condition with severe effects on health and wellbeing, imposes a substantive economic burden, and has no known cure (Lockwood et al., 2002). Up to 50% of adults report to have experienced transient tinnitus following noise exposure, while 5–15% of people living in industrialized societies suffer from chronic tinnitus with negative effects on their quality of life (Gallus et al., 2015; Bhatt et al., 2016). Hearing loss is a common cause of tinnitus and is experienced by up to 86% of adult cochlear implant (CI) candidates (Quaranta et al., 2004).

Knowledge of the pathophysiological mechanisms that trigger and maintain chronic tinnitus is one of the major challenges of tinnitus research whose ultimate purpose is to find a cure (Haider et al., 2018). Peripheral lesions, including loss of hair cells, dysregulated endocochlear potential, and cochlear spontaneous overactivity, could explain the temporary tinnitus occurring immediately after an acute noise trauma (Norena and Eggermont, 2003). In contrast, the finding that bilateral auditory nerve sectioning does not always eliminate tinnitus (Pulec, 1984) suggests that a peripheral lesion is not sufficient to maintain tinnitus and rather represents the trigger of a cascade of neuroplastic changes involving retro-cochlear auditory structures (Henry et al., 2014; Fetoni et al., 2015). Neural reorganization can occur within multiple levels of the central auditory pathway, including the dorsal cochlear nucleus, ventral cochlear nucleus, and inferior colliculus of the brainstem, whose hyperactivity resulting from the downregulation of inhibitory signals has been extensively studied in tinnitus models (Mulders and Robertson, 2009; Zeng et al., 2009). In addition, the medial geniculate body of the thalamus, a major gate of sensory signals to the cortex, the limbic system (Rauschecker et al., 2010), and the primary auditory cortex itself were deemed centers of tinnitus due to the reorganization of the tonotopic map (Eggermont and Roberts, 2004; Knipper et al., 2013; Noreña, 2015). Increasing evidence shows that auditory deprivation leads to chronic subjective tinnitus caused by the overrepresentation of adjacent cortical areas with similar characteristic frequency, known as “edge frequencies,” due to the lack of lateral inhibition phenomena (Eggermont and Roberts, 2004). Thus, maladaptive plastic changes in the central auditory pathways may be involved in maintaining tinnitus in a sort of “vicious circle” (Fetoni et al., 2015). Therapeutic implications are significant since it was first supposed that only peripheral tinnitus could be masked by sounds as opposed to tinnitus powered by central generators (Haider et al., 2018). Hence, the pathogenesis is still unclear and multiple mechanisms at various levels of the auditory system are likely to concur.

To date, only a few tinnitus treatments are available, but there is no pharmacological approach approved by the major drug agencies. Electrical stimulation delivered both transcutaneously (Steenerson and Cronin, 2003; Aydemir et al., 2006) and transtympanically (Konopka et al., 2001; Rubinstein et al., 2003; Di Nardo et al., 2009) has been proposed as a promising approach to suppress peripheral tinnitus. It has been suggested that electric current could act both at a presynaptic level, with the reduction in the spontaneous release of neurotransmitters from inner hair cells (Konishi et al., 1970), and with a postsynaptic mechanism by reducing the opening of voltage-gated sodium channels, with a direct effect on the membrane potential of the cochlear fibers (Shepherd and Javel, 1999). On this basis, central tinnitus, which becomes independent of cochlear residual spontaneous activity, should not respond to this treatment (Noreña et al., 2015). Interestingly, in the experimental model, brain stimulation induced by the anodal transcranial direct current affects the structural plasticity of the auditory cortex and compensates for the effects of sensory deprivation following cochlear damage by increasing dendritic spine numbers and rearranging synaptic networks (Paciello et al., 2018) in the primary auditory cortex. Therefore, attempts have been made to relieve chronic intractable tinnitus by delivering different electrical stimuli directly to the auditory cortex (De Ridder et al., 2006; Seidman et al., 2008) with doubtful results and many possible side effects related to the invasiveness of the method.

Nowadays, the different methods used and the uncertainty of clinical efficacy have made it impossible to guide technological development toward a real therapy (Assouly et al., 2021). Considering this scenario, the intracochlear electrical stimulation *via* CI could represent a putative approach to tinnitus treatment. Moreover, the evaluation of the effects of electrical stimulation should improve the general knowledge of tinnitus mechanisms. Beneficial effects on tinnitus have been previously reported in many CI users (Quaranta et al., 2004; Di Nardo et al., 2007b; Ramakers et al., 2015; Peter et al., 2019), but the cause is still not understood: experimental studies on animals suggest that CI can restore a certain degree of normal discharge in the cochlear nerve through the inhibition of spontaneous activity or even a reflex increase in microcirculation in the auditory pathway (Runge-Samuelson et al., 2004). In contrast, the long-term stable effects of the CI on tinnitus would require the reorganization of the central auditory cortex. The masking theory, which is secondary to hearing relief, is unlikely since most patients report persistent improvement of their tinnitus even at night when CI is off (Quaranta et al., 2004). It has been proposed that auditory stimulation could reverse the tinnitus-related central changes, but the presence of degenerated cochlear fibers hinders the process of restoring the pre-hearing loss distribution of sensory inputs to the auditory centers (Noreña, 2015). In addition, recent attempts to optimize intracochlear electrical stimulation to reduce tinnitus have not led to clear results (Arts et al., 2015, 2016), suggesting that the characteristics of the stimulus for tinnitus reduction are highly subject-specific. Furthermore, the most effective target of intracochlear electrical stimulation was not investigated by previous studies.

Assuming that the stimulation of a limited area of the cochlea involved in triggering and maintaining chronic tinnitus could improve tinnitus in CI users, as opposed to the non-specific stimulation of the entire cochlea, we aimed to further investigate the intracochlear electrical stimulation and identify the electrical channel(s) of the CI array that suppress/attenuate tinnitus.

### Objectives

The major aim of our study was to measure in each patient the variation of the subjectively perceived tinnitus loudness during short-term single-channel stimulation and to find the best-performer channel (i.e., the channel that caused the greatest tinnitus reduction). Furthermore, the correlation between the reduction of tinnitus loudness and the position of the channels inside the cochlea has been evaluated in all patients to define the cochlear regions involved in tinnitus improvement.

We also aimed (i) to compare the impact of short-term single-channel electrical stimulation with the normal functioning of the whole CI in the early phase of activation in terms of tinnitus improvement, (ii) study long-term results of CI on the subjective perception of tinnitus, and (iii) evaluate whether qualitative characteristics of tinnitus (monotone vs. polyphonic tinnitus) could influence the results of short- and long-term intracochlear electrical stimulation on tinnitus loudness.

## Materials and Methods

### Subjects

It was an observational prospective case-crossover study. Subjects were enrolled from January 2021, and the study lasted for 1 year. Before enrollment, all patients received complete and comprehensible information regarding the tests administered and gave their written consent, in agreement with the ethical standards of the Declaration of Helsinki. The study was approved by our institution’s ethics committee under protocol no. 0023756/21.

All candidates for unilateral cochlear implantation surgery at our institution preoperatively underwent an accurate medical history focused on duration, type and cause of hearing loss, and onset and characteristics of tinnitus. A complete audiological evaluation was performed, including otoscopy, tympanometry, and acoustic reflex measurement (Grason Stadler Tympstar), as well as standard pure-tone audiometry, testing conventional frequency ranging from 0.25 to 8 kHz (Amplaid 319 audiometer, Amplaid Inc.) in a double-walled, soundproof room. Preoperative pure tone average (PTA) (average of hearing threshold levels at 500, 1,000, 2,000, and 4,000 Hz) was measured on both ears in all patients. All patients had bilateral sensorineural HL, which was severe to profound (PTA > 70 dB HL) in the worst ear and slight to profound in the other ear (Table 1). Inclusion criteria were as follows: age ≥ 18 years; chronic (at least 6 months) tinnitus perceived in the worst hearing-impaired ear; intracochlear placement of the implant through the (extended) round window membrane; and the ability to read, understand, and fill in the assigned questionnaires and sign an informed consent form.

**TABLE 1:** Patients’ demographic characteristics, causes, and duration of hearing loss (HL) and preoperative audiometric data.

Patient	Sex	Age (yrs)	Hearing Loss (HL) cause	HL, duration (yrs)	PTA R (db)	PTA L (db)
N1	Female	53	Ménière’s disease	16	45	114
N2	Female	65	Otosclerosis	43	68	120
N3	Male	68	Idiopathic	18	120	99
N4	Male	62	Idiopathic	53	84	103
N5	Female	22	Sudden HL	10	102	99
N6	Male	58	Idiopathic	58	115	115
N7	Female	68	Otosclerosis	50	120	114
N8	Female	58	Cogan syndrome	24	63	120
N9	Male	53	Otosclerosis	30	102	102
N10	Female	56	Idiopathic	13	120	120
N11	Male	20	CMV	16	120	120
N12	Female	59	Iatrogenic (aminoglycosides)	46	93	101
N13	Male	55	Auditory neuropathy	13	67	77
N14	Female	46	Hereditary genetics	10	62	72
N15	Male	64	Otosclerosis	19	68	91
N16	Female	47	Idiopathic	20	120	106
N17	Female	38	Idiopathic	14	76	76
N18	Female	63	Otosclerosis	30	91	112
N19	Female	49	Sudden HL	30	84	93
N20	Female	80	Otosclerosis	30	107	120
N21	Male	40	Otosclerosis	10	99	75

*PTA, pure tone average (average of hearing threshold levels at 500, 1,000, 2,000, and 4,000 Hz); R, right ear; L, left ear.*

Patients with tinnitus onset after surgery were not enrolled. Other exclusion criteria were pulsatile tinnitus, congenital malformation of the auditory system detected with preoperative CT and MR imaging of the inner ear and brain, history of vestibular schwannoma, active middle ear disease, and complications during or after surgery (i.e., flap necrosis, improper electrode placement, facial nerve problems, infection, facial nerve stimulation, vertigo). Cases with incomplete/difficult insertion of the array into the cochlea were also excluded. The insertion of the CI in the cochlea was demonstrated in all patients with intraoperative X-ray static fluoroscopy (Garaycochea et al., 2020) to avoid possible extracochlear array misplacement (e.g., semicircular canal, vestibule, middle ear), tip rollover, kinking, or lopping. All patients underwent the intraoperative electrophysiological test to verify the neural response from all the CI’s electrical channels.

Patients with a history of psychiatric disorders, depression, and use of antidepressant treatments, as well as patients affected with neurodegenerative diseases, especially Alzheimer’s and Parkinson’s diseases, were also excluded from the study.

### Study Design

#### Study Questionnaires

All patients underwent the assessment of tinnitus characteristics by using self-administered questionnaires as follows:

- *The Tinnitus Characteristics Questionnaire for CI recipients* used by Wang et al. (2017) was translated into Italian and administered immediately before surgery to define the qualitative characteristics of hearing loss and tinnitus (cause, laterality, grading, duration), typology (subjective, objective), year of onset, localization (bilateral, unilateral right or left, central), components (monotone or polyphonic, intermittent, or continuous), subjectively defined type of tinnitus (cicadas, roar, crackle, rain, wind, hum, whistle, music), and aspect of greatest influence on daily life (sleep, hearing, emotion, work, memory).

- *The Tinnitus Handicap Inventory (THI)* in its validated Italian version (Monzani et al., 2008) was administered before surgery, 1 month (immediately before CI activation), 6 months, and 1 year after surgery to study long-term CI effect on tinnitus. THI was administered according to the model proposed by Newman (Newman et al., 1996) and graded according to the McCombe grading system (McCombe et al., 2001). The THI questionnaire is composed of 25 questions, each with three quantifiable answers (yes = 4, sometimes = 2, no = 0). The final total score (0–100) defines the degree of subjective perception of tinnitus in the last week: grade 1, very slight (THI score 0–16); grade 2, mild (THI 18–36); grade 3, moderate (THI 38–56); grade 4, severe (THI 58–76); grade 5, catastrophic (THI 78–100).

- *Visual Analog Scale (VAS)*, which is used in clinical research to measure the intensity of symptoms, was administered to patients to assess perceived tinnitus loudness. Patients were asked to rate the perceived loudness of their tinnitus on a horizontal scale oriented from right to left, from 0 (inaudible) to 10 (loud like never before). VAS was administered before surgery (L*^S^*); 4 weeks after surgery (immediately before CI activation) to define the baseline loudness of tinnitus (L^0^); on the day of activation during the experimental procedure *via* the single electrical channel stimulation (L^1^); and on the day of activation, 30 min after the whole CI was first turned on (L^2^). This scale expresses the subjective perception of tinnitus, namely, absent (0–1), mild (2–3), moderate (3–6), severe (6–8), and very serious (9–10).

#### Study Procedures

Enrolled patients underwent the following procedures:

- *CI activation and mapping:* CI was activated 4 weeks after surgery. All channels were sequentially activated. The maximum comfort level (C-level) and mean threshold level (T-level) were determined based on subjective responses. The full CI frequency range was distributed to the different electrical channels in accordance with the CI manufactures’ standards that are based on the Greenwood’s function (Greenwood, 1961).

- *Experimental procedure – single electrical channel stimulation*: The short-term effect of electric current on tinnitus was evaluated during the stimulation of the different electrical channels with the C-level, for 10 s, one by one, starting from the cochlear apex toward the base, with a recovery time of 30 s between one channel and the next. The basic stimulation parameters for each brand are shown in Table 2. For each channel, the patients were asked to rank their perceived tinnitus during electrical stimulation on the VAS. The level of tinnitus reduction was expressed in percentiles relative to the baseline loudness and calculated using the following equation:

R=T(L-0-L)1×100/L0

where R*^T^* represents the amount of tinnitus reduction, and L^0^ represents the baseline perceived tinnitus loudness before stimulation. The loudness ranked on the VAS during stimulation is denoted as L^1^. A tinnitus reduction of 0% corresponds to no change in perceived tinnitus loudness, while positive values correspond to tinnitus reduction and negative values correspond to a worsening of tinnitus.

**TABLE 2:** Detection of cochlear regions most involved in the attenuation/suppression of tinnitus: electrical channel with the greatest attenuation capacity on tinnitus in each patient [suppressive channel (SC)]; minimum current level suppressing tinnitus (MCLT) (μA).

Patient	SC	Frequency	MCLT (μA)	CI model	Electrode length	Strategy of stimulation	Frequency of stimulation	Pulse width
N1	22	125–313	215,4	Cochlear CI612 Peri-modiolar	19mm	MP3000	900	25
N2	7	3063–3563	244,4	Cochlear CI512 Peri-modiolar	19mm	MP3000	900	25
N3	Nf	/	/	Cochlear CI632 Peri-modiolar	17mm	ACE	900	25
N4	Nf	/	/	MED-EL Mi1200Flex28 Lateral-wall	28mm	FS4	1237	25.42–30.42
N5	17	563–688	735,5	Cochlear CI512 Peri-modiolar	19mm	ACE	900	25
N6	4	491–710	368	MED-EL Mi1200Flex28 Lateral-wall	28mm	FS4	1660	12.08–17.92
N7	11	1813–2063	235,7	Cochlear CI512 Peri-modiolar	19mm	ACE	900	25
N8	22	125–313	344,5	Cochlear CI24RE CA Peri-modiolar	19mm	ACE	900	25
N9	22	125–313	613,9	Cochlear CI512 Peri-modiolar	19mm	ACE	900	25
N10	6	3563–4063	348,9	Cochlear CI422 Lateral-wall	25mm	ACE	900	25
N11	22	125–313	204	Cochlear CI512 Peri-modiolar	19mm	ACE	900	25
N12	22	125–313	443,5	Cochlear CI612 Peri-modiolar	19mm	MP3000	900	25
N13	11	1813–2063	215,4	Cochlear CI512 Peri-modiolar	19mm	ACE	900	25
N14	Nf	/	/	AB Hires 90KJ Lateral-wall	20mm	HRes Optima-S	1258	53
N15	Nf	/	/	Cochlear CI422 Lateral-wall	25mm	ACE	900	25
N16	22	125–313	560,9	Cochlear CI24RE CA Peri-modiolar	19mm	ACE	900	25
N17	1	100–198	397	MED-EL Mi1200Flex28 Lateral-wall	28mm	FS4	1277	17.08–33.75
N18	6	3563–4063	485,5	Cochlear CI612 Peri-modiolar	19mm	ACE	900	25
N19	19	563–688	309	Cochlear CI612 Peri-modiolar	19mm	ACE	900	25
N20	Nf	/	/	Cochlear CI512 Peri-modiolar	19mm	ACE	900	25
N21	Nf	/	/	Cochlear CI512 Peri-modiolar	19mm	ACE	900	25

*CI, cochlear implant model; nf, not found.*

According to Arts et al. (2016), R*^T^* was considered significant when > 50%, and it was graduated as follows, namely, complete suppression of tinnitus (90% < R*^T^* ≤ 100%), relevant attenuation (50% < R*^T^* ≤ 90%), mild attenuation (10% < R*^T^* ≤ 50%), and no effect on tinnitus (R*^T^* ≤ 10%).

R*^T^* was measured in each patient for each channel of the CI. The attenuation/suppression effect of electrical stimulation on tinnitus perception was considered “selective” when it was possible to find an electrical channel with a significantly higher tinnitus-reducing effect than the other channels [suppressive channel (SC)]; otherwise, the effect was considered “non-selective.” The frequency ranges assigned to the SCs were considered to establish their location inside the cochlea. Multiple comparisons between mean L^0^ and mean L^1^ measured for each frequency range have been done to find the cochlear area most affected by tinnitus improvement in our sample.

Once the channel with the highest attenuation effect on tinnitus was identified, the intensity of the current was gradually reduced until tinnitus occurred again in the patient to identify the minimum current level suppressing tinnitus (MCLT) (μA).

- *Pitch-matching procedure:* The variability in the cochlear size and, above all, the different insertion depths of the array can create a mismatch between the default frequencies assigned to each channel and their actual location in the cochlea (Di Nardo et al., 2007a,Di Nardo et al., 2010). Patients with hearing residues in the non-implanted ear underwent the procedure of mismatch evaluation (Di Nardo et al., 2011), consisting of the pitch comparison between the electrical and acoustic stimuli sent independently to the two ears. Each pitch comparison trial consisted in first stimulating an electrical channel of the CI with a 5 s electric stimulus, followed by a 5 s acoustic pure tone presented to the other ear to find the corresponding frequency. The patient was asked to verbally report the tone sent to the other ear as higher, lower, or similar in pitch. The frequency of the acoustic stimulus was adaptively changed by 1/12th of an octave up or down, depending on the subject’s response. A minimum of three matching attempts were conducted. Both stimuli could be presented in a random order where either electrode stimulus or acoustic tone was presented first. The procedure can be repeated for all channels, but for the purpose of the study it was sufficient to find the SC pitch. The procedure requires a good degree of auditory rehabilitation; therefore, it was performed 6 weeks after CI activation.

### Statistical Analysis

Statistical analysis was performed using Microsoft Excel (Microsoft Corporation, Redmond, WA, United States). Continuous values, such as the THI score, are expressed as mean ± standard deviation (SD). Qualitative variables were summarized with absolute and percentage frequency tables.

The primary objective of this study was achieved by calculating the greatest reduction of the perceived loudness of tinnitus (R*^T^*) on a VAS in each patient during short-term single-channel stimulation, with the abovementioned formula [R*^T^* = (L^0^ – L^1^) × 100/L^0^], considering R*^T^* to be significant when it was > 50%. The electrical channel with the greatest R*^T^* compared to the other channels was found in each patient. Multiple *t*-test comparisons between mean L^0^ and mean L^1^ measured for each frequency range have been done to find the cochlear area most affected by tinnitus improvement in our sample. The secondary objective, consisting of the comparison between continuous VAS scores obtained during single-channel stimulation and after CI activation, was achieved using the *t*-test for paired data. Long-term CI results on tinnitus perception were measured through THI before surgery, 1 month, 6 months, and 1 year after surgery; mean THI score obtained for each time point was compared to the preoperative score using *t*-test for paired data. The intergroup comparison considering the qualitative characteristics of tinnitus (incidence of monotone vs. polyphonic tinnitus) was performed using the chi-square test. The results were considered significant for *p* values < 0.05.

## Results

### Patients

A total of 21 adult patients suffering from profound sensorineural hearing loss (HL) and subjective chronic tinnitus undergoing unilateral cochlear implantation were finally included (Table 1): 13 women (61.9%) and 8 men (38.1%) aged between 20 and 80 years (mean: 53.5 ± 15). Table 1 summarizes the patients’ demographic characteristics, side of deafness, grading and causes of hearing loss, as well as preoperative audiometric data.

The implanted devices used were Cochlear (Cochlear Ltd, Melbourne, Australia) (17/21), MED-EL (MED-EL Corp., Innsbruck, Austria) (3/21), and Advanced Bionics (Advanced Bionics LLC, Valencia, CA) (1/21). Table 2 describes the implanted devices, characteristics of the electrodes, strategy, pulse width, and frequency of stimulation used.

### Tinnitus Characteristics

The patients in question had been suffering from chronic tinnitus for an average of 18.5 (± 13.6) years. The questionnaire developed by Wang et al. (2017) regarding the characteristics of tinnitus showed that 8/21 (38.1%) patients had unilateral tinnitus (ipsilateral to the CI) while 13/21 (61.9%) patients had bilateral tinnitus.

Tinnitus was described as monotone in 16/21 (76.2%) patients and polyphonic in 5/21 (23.8%). A total of 21/21 (100%) patients were affected by continuous tinnitus.

**FIGURE 1:**
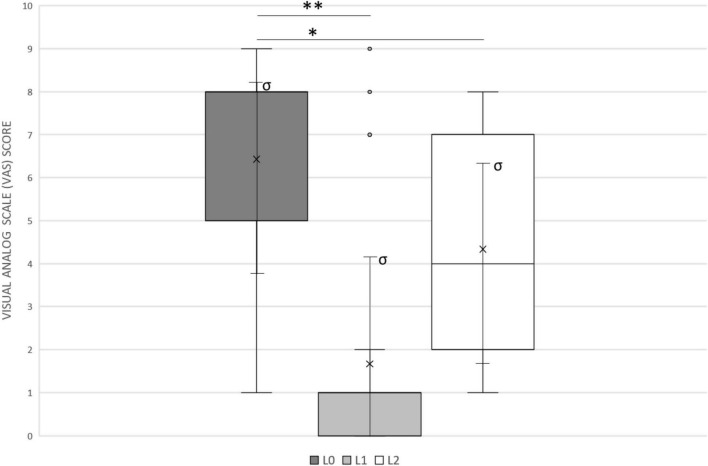
The Visual Analog Scale (VAS) was used to assess tinnitus modification. Patients were asked to rate the perceived loudness of their tinnitus on a numeric scale, from 0 (inaudible) to 10 (loud like never before). L^0^, basal loudness of tinnitus (mean 6.4 ± 2.4); L^1^, minimal loudness of tinnitus at 10 s single-channel stimulation (mean 1.7 ± 2.7); L^2^, loudness of tinnitus after 30 min of whole CI activation (4.3 ± 2.5). Median values are displayed as a horizontal line, mean values as x, standard deviation as σ. We used *t*-test for statistical analysis. ^∗∗^*p* < 0.01, ^∗^*p* < 0.05.

Tinnitus loudness assessed using the VAS before surgery (L*^S^* = 6.5 ± 2.5) and 4 weeks after surgery (before the activation) (L^0^ = 6.4 ± 2.4) did not change significantly (*p* > 0.05). Also, 30 min after CI activation and mapping (CI turned on), the mean VAS value (L^2^) was 4.3 ± 2.5. CI activation induced a significant reduction of subjective perception of tinnitus as measured by the VAS (L^0^ vs. L^2^; *p* = 0.0095), even in the early stage of stimulation (Figure 1). Specifically, 10/21 (47.6%) patients reported a significant improvement in VAS (R*^T^* > 50%) after CI activation, whereas in 2/21 (9.5%) the tinnitus worsened when the CI was turned on. In 2/21 (9.5%) patients, CI activation had no effect on subjective perception of tinnitus.

### Short-Term Effect of Single-Channel Stimulation and Detection of Cochlear Regions Involved in the Attenuation/Suppression of Tinnitus

**FIGURE 2:**
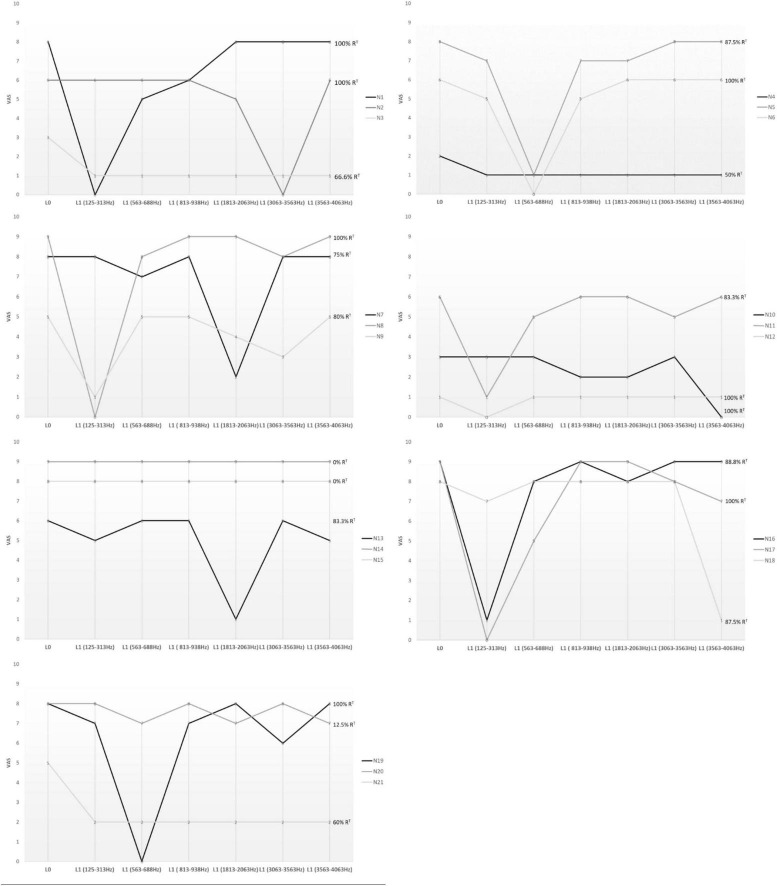
The Visual Analog Scale (VAS) was used to assess tinnitus modification. Patients were asked to rate the perceived loudness of their tinnitus on a numeric scale, from 0 (inaudible) to 10 (loud like never before), during short-term single-channel stimulation. The figure shows the cochlear regions involved in each patient. Only the frequency bands corresponding to the best-performer channels are depicted in the figure, not the entire cochlear tonotopy. L^0^, basal loudness of tinnitus; L^1^, loudness of tinnitus at 10 s single-channel stimulation; R*^T^*, greatest percentage of tinnitus reduction.

The experimental procedure showed that 8/21 patients (38%) experienced complete suppression of tinnitus (90% < R*^T^* ≤ 100%) through the activation of a specific channel (SC); 7/21 (33.3%) experienced a significant but not full attenuation of tinnitus (50% < R*^T^* ≤ 90%) with the SC (Figure 2).

In 4/21 patients, the tinnitus effect was non-selective: 3/21 (14.3%) reported a significant (50% < R*^T^* ≤ 90%) but non-selective attenuation, 1/21 (4.7%) reported a mild and non-selective attenuation (10% < R*^T^* ≤ 50%). 2/21 (9.5%) had no tinnitus effect (R*^T^* ≤ 10%). No one reported worsened tinnitus symptoms during or after stimulation.

Considering the mean baseline loudness L^0^ 6.4 ± 2.4, the mean VAS score reported by patients during the experimental procedure (L^1^) was 1.7 ± 2.7, with a significant difference (*p* = 0.003) and a mean reduction in loudness of 74.9%. Consequently, short-term targeted stimulation was more effective than the stimulation with the whole CI in our sample (L^1^ vs. L^2^, *p* = 0.0002) in the early phase of activation (Figure 1).

A total of 15/21 patients (71.4%) had a significant R^*T*^ *via* the stimulation of a specific channel, reporting a mean L^1^ of 0.4 ± 2.0 (L^0^ vs. L^1^, *p* = 0.0001), with an average reduction in loudness of 93%. Thus, for most patients, tinnitus improvement was caused by the stimulation of a narrow area of the cochlea. The characteristics of the electrodes (12/16 perimodiolar-positioned and 3/6 lateral wall) and their length, as shown in Table 2, did not influence tinnitus perception. We identified the best-performer channel as reported in Figure 2 and Table 2. Namely, for Cochlear implants (17/21), the selective stimulation on channel number 22 (188–313 Hz band) significantly reduced the tinnitus perception in 6/17 (35.3%) patients, Cochlear number 19 (563–688 Hz) in 1/17 (5.9%), Cochlear number 17 (813–938 Hz) in 1/17 (5.9%), Cochlear number 11 (1,813–2,063 Hz) in 2/17 (11.8%), and Cochlear number 6 (3,563–4,063 Hz) in 3/17 (17.6%). For MED_EL CIs (3/21), channel number 1 (70–170 Hz) reduced tinnitus in 1/3 (33.3%) patients; MED-EL channel number 4 (491–710 Hz) in 1/3 (33.3%).

Seven patients with hearing residues in the non-implanted ear were able to perform the pitch-matching test. In three patients (N1, N2, N5), the pitch heard during the procedure did not fall within the frequency range empirically assigned to the SC: patient N1 (channel 22 – 125 Hz); patient N2 (channel 6 – 3,400 Hz); and patient N5 (channel 17 – 625 Hz). Table 3 shows the results of the pitch-matching procedure.

**TABLE 3:** Results of the pitch matching procedure.

Patient	SC	Frequency band	Real pitch
N1	22 Cochlear	188–313 Hz	**125 Hz**
N2	6 Cochlear	3563–4063 Hz	**3400 Hz**
N5	17 Cochlear	813–938 Hz	**625 Hz**
N8	22 Cochlear	188–313 Hz	225 Hz
N13	11 Cochlear	1813–2063 Hz	2000 Hz
N17	1 MED-EL	100–198Hz	170 Hz
N19	19 Cochlear	563–688 Hz	625 Hz

*In three patients (N1, N2, N5), the actual pitch measured (bold values) did not fall 924 within the frequency range empirically assigned to the suppressive channel (SC).*

When considered together, our results showed that the best-performer channels fell within the following frequency bands: 125–313 Hz in 7/15 (46.6%); 563–688 Hz in 3/15 (20%); 1,813–2,063 Hz in 2/15 (13.3%); 3,063–3,563 in 1/15 (6.6%); and 3,563–4,063 Hz in 2/15 (13.3%) (Figure 2 and Table 2). Based on cochlear tonotopy, in 10/15 (66.6%) patients the SC was found in its apical turn, within 1,000 Hz, whereas in 5/15 patients (33.4%) it was found within 4,000 Hz.

Comparing mean L^0^ with mean L^1^ measured for each frequency band, the one corresponding to 125–313 Hz resulted as the cochlear area most affected by tinnitus improvement in our sample (*p* = 0.0074).

To assess the intensity of electric current necessary to suppress tinnitus, for each SC, the MCL value was identified: it was on average 388.5 ± 173.4 μA. For the different frequency bands, 381 μA (125–313 Hz); 522.2 μA (563–688 Hz); 368 μA (813–938 Hz); 225.5 μA (1,813–2,063 Hz); 244.4 μA (3,063–3,563 Hz); 417.2 μA (3,563–4,063 Hz) (Table 2). We observed that the intensity of electric current was not related to the baseline loudness of tinnitus measured with the VAS (Pearson *R* = 0.03), nor to the THI (Pearson *R* = 0.03). Conversely, it was shown to be independent of the subjective perception of tinnitus. Table 2 shows the results of the test for each patient.

All patients undergoing the experimental procedure were retested to verify that the best-performer channel described in the early phase of activation was the same after 2 weeks. The repeatability rate of our experimental procedure, calculated as the percentage of patients whose measures were found to be the same after 2 weeks, was 95%, obtaining the same results in 20/21 patients. The results only changed between the two settings in one patient (N5) [channel number 21 (range 313–438 Hz) instead of channel 17].

### Tinnitus Effect on Daily Life and Long-Term Effect of Intracochlear Electrical Stimulation

When asked to complete the Qian Wang questionnaire, 8/21 (38%) patients reported that the most serious impairment was related to quality of sleep, 6/21 (28.6%) to hearing and speech perception, 4/21 (19%) to emotional state, 2/21 (9.5%) to memory and ability to concentrate, and 1/21 (4.7%) to work activity.

The mean preoperative THI was 39.7 ± 27.2. Four weeks after surgery, the mean THI was 40.3 ± 26.7 (before activation), with no statistically significant difference (*p* > 0.05); in 6/21 (28.6%) patients, the tinnitus worsened in the immediate postoperative period showing an increased grading class, while 5/21 (23.8%) patients reported tinnitus improvement following surgery with a decreased grading class.

**FIGURE 3:**
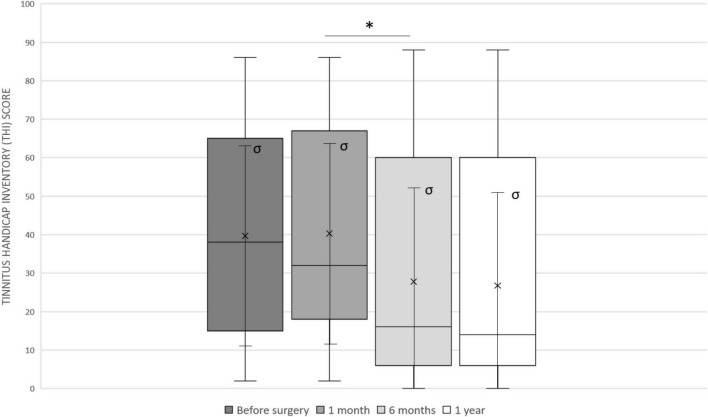
The Tinnitus Handicap Inventory (THI) trend in our sample: before surgery (mean 39.7 ± 27.2), 4 weeks after surgery (mean 40.3 ± 26.7), after 6 months (mean 27.8 ± 29.5) and after 1 year (mean 26.7 ± 28.9). Median values are displayed as a horizontal line, mean values as x, standard deviation as σ. We used *t*-test for statistical analysis. ^∗^*p* < 0.05.

Six months after activation, the THI was 27.8 ± 29.5 with a statistically significant difference compared to the preoperative value (*p* = 0.0325). Mean THI at 1 year (26.7 ± 28.9) was also significantly higher (*p* = 0.0244). There were no significant changes between 6 months and 1 year. Figure 3 shows the trend of THI score in our sample.

### Qualitative Characteristics of Tinnitus and Long-Term Results (Intergroup Comparison)

The qualitative characteristics of tinnitus were analyzed and compared between the group of patients who had a best-performer channel (15/21), the patients who had a non-selective attenuation (4/21), and the two patients who had no tinnitus changes during the experimental procedure (2/21).

The four patients who had a non-selective attenuation were compared to patients with selective suppression (Table 4). They had a more recent onset of tinnitus (11 vs. 20.8 years, *p* < 0.05), a higher incidence of subjectively defined polyphonic tinnitus (3/4 – 75% vs. 0/15 – 0%, *p* < 0.05), a lower baseline VAS with CI off (4.5 vs. 6.6, *p* < 0.05), and with CI on (2.5 vs. 4.5, *p* < 0.05), a lower THI pre-implantation (36.5 vs. 40.6, *p* > 0.05), on the day of CI activation (23 vs. 44.6, *p* < 0.05), and 1 year after surgery (18.5 vs. 30.5, *p* < 0.05).

**TABLE 4:** Intergroup comparison.

Patients	Polyphonic tinnitus	mean VAS (CI off – L0)	mean VAS (CI on – L2)	mean THI pre-op.	mean THI 1 year
Selective attenuation of tinnitus 15/21	0%	6.6	4.5	40.6	30.5
Non-selective attenuation of tinnitus 4/21	75%*	4.5*	2.5*	36.5	18.5*
No effect on tinnitus 2/21	100%*	8*	6.5*	72*	40*

*Statistically significant difference compared to patients with specific attenuation on tinnitus, p < 0.05.

The two patients who did not report changes during the experimental procedure, compared to patients having selective suppression, had subjectively defined polyphonic tinnitus (2/2 – 100% vs. 0/15 – 0%, *p* < 0.05), a higher VAS with CI off (8 vs. 6.6, *p* < 0.05), and on (6.5 vs. 4.5, *p* < 0.05), a higher THI 1 year after surgery (40 vs. 30.5, *p* < 0.05).

Therefore, patients in whom it was possible to obtain a short-term suppression of tinnitus with a specific channel had a subjectively defined monotone tinnitus of longer duration and a greater subjective perception of tinnitus. On the other hand, the non-specific attenuation was related to better long-term results measured with THI, possibly due to a lower perceived loudness and the possibility to attenuate the tinnitus by stimulating the entire cochlea rather than selectively stimulating a single electrical channel, which is the way the CI normally works. Patients who reported no changes in tinnitus perception during the experimental procedure also had poor long-term results with 1-year THI.

The research had no missing data for any of the measured variables.

## Discussion

Our results showed that the intracochlear electrical stimulation significantly reduced the subjective tinnitus perception, in agreement with previous reports (Quaranta et al., 2004; Ramakers et al., 2015; Peter et al., 2019). In detail, we observed a significant improvement of tinnitus handicap severity 6 months after CI activation as measured with the THI (from 39.7 ± 27.2 to 27.8 ± 29.5; *p* = 0.0325). It is reasonable to expect that tinnitus can be partially alleviated by increasing auditory stimulation, considering the current consensus that chronic tinnitus is the result of maladaptive plasticity of the auditory cortex consequent to sensorial deprivation (Engineer et al., 2011). Increasing evidence indicates that electrical current is a promising treatment itself, independently of auditory stimulation (Shepherd and Javel, 1999; Konopka et al., 2001; Rubinstein et al., 2003; Steenerson and Cronin, 2003; Aydemir et al., 2006; De Ridder et al., 2006; Seidman et al., 2008; Di Nardo et al., 2009; Noreña et al., 2015; Paciello et al., 2018). To define which stimulation patterns should be optimized for tinnitus relief, a deeper understanding of the mechanisms involved in tinnitus suppression is needed (Assouly et al., 2021). CI represents the main tool to investigate the effects of intracochlear electric current in patients with chronic tinnitus.

The experimental animal models revealed that the mechanism behind the efficacy of electric current on tinnitus was the reversal of the reorganization of the auditory structures involved in chronic tinnitus, rather than the shift in attention from tinnitus to external sounds (Noreña et al., 2015; Paciello et al., 2018). This mechanism, based on the effectiveness of electrical hearing, could explain the evidence of persistent tinnitus reduction in many patients after the CI stimulation is turned off (Quaranta et al., 2004). Interestingly, previous studies showed short-term tinnitus suppression *via* CI, independently of environmental sound stimulation, by interfering with the fitting software (Arts et al., 2015, 2016). Accordingly, it was suggested that the coding of environmental sounds is not required to reduce tinnitus.

However, it remains unclear whether the beneficial results on tinnitus observed with CI, in a percentage of patients that varies from 25 to 72% (Ramakers et al., 2015), depend on the undifferentiated stimulation of the whole cochlear partition or on a spatially limited alteration induced by electric current.

Therefore, we aimed to evaluate whether the single-channel stimulation of CI could improve tinnitus perception and whether it was possible to identify a region in the cochlea whose stimulation would cause beneficial effects on tinnitus. To exclude that the effects on tinnitus perception could depend on electrical hearing and avoid any possible alteration of baseline tinnitus characteristics induced from electric current, the experimental procedure was performed in the early phase of CI activation. We introduced a new procedure to be combined with CI mapping in patients affected by chronic tinnitus, showing that electric current delivered on a single channel of the CI for 10 s constituted the “trigger” of tinnitus suppression in 71.4% of our sample [L^0^ (6.4 ± 2.4) vs. L^1^ (1.7 ± 2.7), *p* = 0.003]. Patients in whom it was possible to identify a best-performer channel reported a significant reduction in their tinnitus-perceived loudness as measured with the VAS during stimulation (L^1^ = 0.4 ± 2.0, *p* = 0.0001), with an average reduction in loudness of 93%. The results suggest that tinnitus subjective perception could be partially or totally alleviated by an electrical stimulus targeted to a limited region of the cochlea rather than to the entire cochlear duct.

Furthermore, short-term targeted stimulation was more effective than stimulation involving the whole CI in the early phase of activation [L^1^ (1.7 ± 2.7) vs. L^2^ (4.3 ± 2.5), *p* = 0.0022]. The possibility to find a more sensitive area to tinnitus attenuation even in postverbal deaf patients with an almost completely compromised cochlea suggests that tinnitus probably arises from a limited area of the basilar membrane and secondarily involves other relays of the auditory pathway. For this reason, we hypothesized that the stimulation of a specific region of the damaged cochlea could be the most involved in long-term tinnitus improvement and in the reversal of cortical reorganization maintaining chronic tinnitus (Haider et al., 2018).

Another important challenge was to understand whether and how the location of the electrical channels along the CI array and their corresponding frequency were related to the reduction of tinnitus. Several concerns affect the knowledge of the exact tonotopic position of the channels inside the cochlea, including the different electrodes used, their different insertion depths, and the variable tonotopic organization in damaged cochleae (Rak et al., 2020; Niu et al., 2021). Consequently, it is very difficult to determine exactly the regions stimulated by the channels, except in patients with asymmetric hearing thresholds. Despite these issues, the exact pitch of the best-performer channel was identified in seven patients who underwent the pitch-matching procedure, showing that the most effective stimulation frequencies were those at the apex of the cochlea, from 125 to 313 Hz (*p* = 0.0074). This finding seems to run counter to the experimental model of tinnitus consequent to acoustic trauma or ototoxic drugs and suggests that in most cases the initial dysfunction is in the basal turn of the cochlea (Ralli et al., 2014; Paciello et al., 2020). However, our patients were affected by severe to profound hearing loss, indicating extensive cochlear damage. Furthermore, since CIs stimulate the spiral ganglion fibers, it is reasonable to assume that their unpredictable number and particularly their distribution also influence the results (Dhanasingh et al., 2020). As it stands, we do not know if the cochlear region with the greatest attenuation capacity on tinnitus is that of initial cochlear damage triggering tinnitus. Considering that cochlear damage was demonstrated to determine the overrepresentation of the edge frequencies in the cortex due to the loss of lateral inhibition phenomena (Eggermont and Roberts, 2004), we hypothesized that the electrical stimulation of the overrepresented area can mask or reverse mechanisms favoring tinnitus. While some studies have localized the tinnitus pitch at the edge of hearing loss, others found that it occurs in the region of maximum hearing loss (Sereda et al., 2011). We were not aware of the real tinnitus pitch in our sample as it was only subjectively defined with the Qian Wang questionnaire because of the severe-to-profound hearing loss of the patients and the impossibility to evaluate it with the audiometry. In addition, this study, by nature, evaluates tinnitus through subjective scores (THI, VAS), by asking participants to rate their tinnitus several times during the experiments, which could have influenced the results. To select a cohort of patients with single-sided deafness or asymmetric thresholds, it should be essential in the future to define whether the tinnitus pitch falls within the frequency band assigned to the suppressive electrical channel and rate the tinnitus loudness using audiometry to further deepen our understanding of the pathophysiological mechanisms of tinnitus. Nevertheless, considering our results, the electrical stimulation of the apical side of the cochlea could represent how to turn off the mechanisms that feed tinnitus.

Coherently with our findings, it has been reported that electrical stimulation in the first 10 mm of the basal part of the scala tympani is not sufficient to reduce tinnitus. Conversely, the stimulation over the complete CI length produced an immediate tinnitus reduction (Punte et al., 2013), suggesting that the stimulation of the basal channels is ineffective for tinnitus attenuation.

Interesting results emerged from the 1-year follow-up with regard to the THI questionnaire: patients with significant tinnitus attenuation achieved through non-selective electrical stimulation, all reporting polyphonic tinnitus, showed lower THI scores compared to CI users with monotone tinnitus in whom the suppressive channel was detected. We assume that the better long-term results in patients with polyphonic tinnitus were related to the effective non-selective attenuation of tinnitus by stimulating along the cochlea through multichannel stimulation. In other words, patients with monotone tinnitus who reported immediate tinnitus attenuation from a single-channel stimulation could not achieve similar long-term results by alternatively stimulating the various frequency bands, which is how the CI normally works. A possible explanation is that a selective continuous amplification of the channel identified by the experimental procedure could be essential in those patients to obtain lasting effects on tinnitus. The long-term CI effects on tinnitus have been assessed by Arts et al. (2015) through the establishment of a sort of “tinnitus implant,” that is, a CI suitably programmed in the different variables considering the parameters subjectively evaluated as more effective in the suppression of tinnitus. These authors, however, do not consider the possibility that a spatially targeted electrical stimulation could offer an additional advantage. Major findings are needed to confirm the correspondence between the stimulation of the cochlear apical turn and tinnitus improvement. Thus, the amplification of the suppressive electrical channel could represent an effective “tinnitus implant” for future clinical use.

In conclusion, the study confirmed the improvement of long-term tinnitus with intracochlear electrical stimulation in CI users. We experimented a procedure focused on the identification and characterization of the suppressive channel obtaining evidence of tinnitus reduction through a targeted electrical stimulation only in patients affected by monotone tinnitus, independently of the tinnitus pitch. We demonstrated that the cochlear apical stimulation is more effective in tinnitus suppression, opening new scenarios for the knowledge of the pathophysiology of tinnitus and future therapeutic applications. The data collected could be helpful for the future development of an implantable stimulator or optimize the CI parameters for tinnitus suppression.

## Data Availability Statement

The raw data supporting the conclusions of this article will be made available by the authors, without undue reservation.

## Ethics Statement

The studies involving human participants were reviewed and approved by Comitato Etico Fondazione Policlinico Agostino Gemelli IRCCS. The patients/participants provided their written informed consent to participate in this study.

## Author Contributions

WD and AF conceived the presented idea and were in charge of overall direction and planning. TD and AT carried out the experiments, collected the data, and contributed to the interpretation of the results. GP supervised the findings of this work. All authors discussed the results and contributed to the final version of the manuscript.

## Conflict of Interest

The authors declare that the research was conducted in the absence of any commercial or financial relationships that could be construed as a potential conflict of interest.

## Publisher’s Note

All claims expressed in this article are solely those of the authors and do not necessarily represent those of their affiliated organizations, or those of the publisher, the editors and the reviewers. Any product that may be evaluated in this article, or claim that may be made by its manufacturer, is not guaranteed or endorsed by the publisher.
